# Correlates of previous couples’ HIV counseling and testing uptake among married individuals in three HIV prevalence strata in Rakai, Uganda

**DOI:** 10.3402/gha.v8.27935

**Published:** 2015-06-08

**Authors:** Joseph K. B. Matovu, Jim Todd, Rhoda K. Wanyenze, Fred Wabwire-Mangen, David Serwadda

**Affiliations:** 1 Department of Community Health and Behavioral Sciences, School of Public Health, Makerere University College of Health Sciences, Kampala, Uganda; 2 Department of Population Health, London School of Hygiene and Tropical Medicine, London, UK; 3 Department of Disease Control and Environmental Health, School of Public Health, Makerere University College of Health Sciences, Kampala, Uganda; 4 Regional Center for Quality of Health Care, School of Public Health, Makerere University College of Health Sciences, Kampala, Uganda

**Keywords:** HIV counseling, testing, married individuals, Rakai, Uganda

## Abstract

**Background:**

Studies show that uptake of couples’ HIV counseling and testing (couples’ HCT) can be affected by individual, relationship, and socioeconomic factors. However, while couples’ HCT uptake can also be affected by background HIV prevalence and awareness of the existence of couples’ HCT services, this is yet to be documented. We explored the correlates of previous couples’ HCT uptake among married individuals in a rural Ugandan district with differing HIV prevalence levels.

**Design:**

This was a cross-sectional study conducted among 2,135 married individuals resident in the three HIV prevalence strata (low HIV prevalence: 9.7–11.2%; middle HIV prevalence: 11.4–16.4%; and high HIV prevalence: 20.5–43%) in Rakai district, southwestern Uganda, between November 2013 and February 2014. Data were collected on sociodemographic and behavioral characteristics, including previous receipt of couples’ HCT. HIV testing data were obtained from the Rakai Community Cohort Study. We conducted multivariable logistic regression analysis to identify correlates that are independently associated with previous receipt of couples’ HCT. Data analysis was conducted using STATA (statistical software, version 11.2).

**Results:**

Of the 2,135 married individuals enrolled, the majority (*n*=1,783, 83.5%) had been married for five or more years while (*n*=1,460, 66%) were in the first-order of marriage. Ever receipt of HCT was almost universal (*n*=2,020, 95%); of those ever tested, (*n*=846, 41.9%) reported that they had ever received couples’ HCT. There was no significant difference in previous receipt of couples’ HCT between low (*n*=309, 43.9%), middle (*n*=295, 41.7%), and high (*n*=242, 39.7%) HIV prevalence settings (*p*=0.61). Marital order was not significantly associated with previous receipt of couples’ HCT. However, marital duration [five or more years vis-à-vis 1–2 years: adjusted odds ratio (aOR): 1.06; 95% confidence interval (95% CI): 1.04–1.08] and awareness about the existence of couples’ HCT services within the Rakai community cohort (aOR: 7.58; 95% CI: 5.63–10.20) were significantly associated with previous receipt of couples’ HCT.

**Conclusions:**

Previous couples’ HCT uptake did not significantly differ by HIV prevalence setting. Longer marital duration and awareness of the existence of couples’ HCT services in the community were significantly correlated with previous receipt of couples’ HCT. These findings suggest a need for innovative demand–creation interventions to raise awareness about couples’ HCT service availability to improve couples’ HCT uptake among married individuals.

Available evidence confirms the role of undiagnosed HIV infections in sustaining the HIV epidemic. In 2006, Marks et al. found that the transmission rate from the HIV status unaware group was 3.5 times that of the aware group after adjusting for population size differences between groups (1). A recent study by Skarbinski et al. found that 30.2% of the estimated 45,000 HIV transmissions that occurred in the United States in 2009 originated from persons who were HIV infected but undiagnosed (2). This problem is more serious in sub-Saharan Africa where less than 50% of individuals living with HIV know of their HIV status (3). Specifically, among HIV-discordant couples that are not aware of their HIV status, the risk of HIV transmission to the uninfected partner ranges between 10 and 20% per annum (4, 5). This risk can be reduced to less than 5% if couples were aware of their HIV status and enrolled into appropriate HIV prevention, care, and treatment programs (6, 7).

Findings from the HIV Prevention Trials Network (HPTN) 052 study show that immediate antiretroviral therapy (ART) enrollment of the HIV-positive partner reduces the risk of HIV transmission to the uninfected partner by 96% (8). A recent study by the Partners Demonstration Project shows that a combination of pre-exposure prophylaxis for the HIV-negative partner and ART for the HIV-positive partner reduces the risk of HIV transmission to the HIV-uninfected partner by 96% (7). These results offer promising opportunities for HIV prevention among known HIV-discordant couples. However, fewer than 30% of couples in sub-Saharan Africa are aware of their HIV sero-status (9, 10), creating a barrier for enrollment into appropriate HIV prevention, care, and treatment services.

Studies show that uptake of couples’ HIV counseling and testing (couples’ HCT) can be hampered by individual, relationship, and socioeconomic factors coupled with fears of the negative social consequences of couples’ HCT (11, 12). In addition, a study conducted in Rwanda and Zambia has shown that lack of knowledge of where to access couples’ HCT services can impact on couples’ HCT uptake (13). However, while these findings offer insights into the reasons for the low uptake of couples’ HCT, there are virtually no studies that have explored couples’ HCT uptake in the context of background HIV prevalence. It is likely that couples in high HIV prevalence settings may be less likely to receive couples’ HCT than their counterparts in low HIV prevalence settings. This is because individuals in high HIV prevalence settings may be more likely to engage in high-risk sexual behaviors; yet, available evidence shows that high-risk individuals are less likely to test together with their partners (9, 11). While these observations may be true, they are yet to be confirmed under any empirical scrutiny. In this paper, we explore the correlates of previous couples’ HCT uptake among married individuals enrolled from different HIV prevalence settings. These data were generated to inform the design and implementation of a cluster-randomized, demand-creation intervention aimed at promoting couples’ HCT uptake among married individuals resident in different HIV prevalence settings in Rakai district, southwestern Uganda.

## Methods

### Study design

This was a cross-sectional study conducted among married individuals to obtain baseline data necessary to inform the design of a cluster-randomized, demand-creation intervention aimed at promoting couples’ HCT uptake among married couples in Rakai, Uganda. The study was implemented in three different settings with differing HIV prevalence levels.

### Study site and HIV prevalence strata

Data were collected from three HIV prevalence strata that were identified for the cluster-randomized, demand-creation intervention within the Rakai community cohort. The Rakai community cohort is a population-based cohort that was established in 1994 for a randomized community intervention trial of STD control for HIV prevention (14) in Rakai district, southwestern Uganda. The cohort consists of 10 study regions, each with approximately 1,500 eligible participants (age range: 15–49 years). Each year, approximately 15,000 consenting individuals aged 15–49 years, resident in the 10 study regions, are administered sociodemographic, behavioral, and health questionnaires. Blood samples are collected for HIV serology and individuals can elect to receive their HIV test results alone or together with their partners. Previous studies in this cohort show that over 80% of the residents have ever received their HIV test results (9, 15) but less than 30% of the tested individuals have ever received their HIV test results as a couple (9). Previous to data collection, the 10 study regions were grouped into low (9.7–11.2%), middle (11.4–16.4%), and high (20.5–43%) HIV prevalence strata based on HIV prevalence data obtained from the ongoing Rakai Community Cohort Study (RCCS) (16). Each stratum had at least three study regions; for this study, one was selected to represent each stratum.

### Study population

The study was conducted among married individuals (aged 15–49 years) who were resident in the three HIV prevalence strata.

### Sample size determination

The sample size for this study was determined based on the number needed to enroll for the cluster-randomized demand-creation intervention. To estimate the sample size for the intervention, we assumed a 35% uptake of couples’ HCT in the intervention communities compared with a baseline of 25% in the standard of care/comparison communities (9). We set two-sided alpha level at 0.05 and assumed a power of 90% to detect differences in the proportion of couples accepting couples’ HCT between the intervention and comparison communities. We used 12 study clusters (4 in each study region) and accounted for cluster design effect using an intra-class correlation of 0.0039 based on an earlier study in Rakai (17). Based on these assumptions, we estimated that we would need to enroll 1,538 individuals in each arm (i.e. intervention and comparison communities) for a total of 3,076 individuals in both arms, after adjusting for non-response rate (out-migration, refusal to participate, and loss to follow-up) estimated at 15% (18). Sample size estimation was done using the *sampsi* and *sampclus* commands in STATA (STATA statistical software, version 11.2).

### Data collection procedures

Data were collected using pilot-tested, structured questionnaires that were administered by same-sex interviewers, in keeping with the same data collection procedures that have been used by the Rakai Health Sciences Program (i.e. the program that runs the Rakai Community Cohort) since the Rakai Cohort was established in 1994. Our experience shows that use of same-sex interviewers can improve collection of sensitive population health data and that respondents find it convenient to interact with same-sex rather than opposite-sex interviewers. Pilot testing of the questionnaire took place in a community outside the designated study regions and helped to improve clarity of the questions to the study team as well as address anomalies in the flow of questions. Data were collected on sociodemographic (e.g. age, education, religious affiliation) and behavioral characteristics (e.g. previous receipt of HCT, HIV status disclosure, marital duration, marital order, and extramarital relations) of all married individuals. All respondents had their data linked to the pre-existing RCCS HIV database to ascertain HIV status (where HIV status information was available) but no fresh blood samples were collected for HIV serology. Data collection took place between November 2013 and February 2014.

### Measurement variables

The primary outcome of the study was previous couples’ HCT uptake. This was defined as self-reported previous receipt of HCT services by both members of a couple at the same sitting. A couple was defined as a man and a woman in a steady sexual relationship, regardless of whether they were married religiously, traditionally, or through the court registrar's office.

Previous receipt of couples’ HCT was assessed by asking respondents if they had ever tested and received their HIV test results with any of their sexual partners, including with their current marital partners. We defined HIV status disclosure as self-reported disclosure of HIV status to any of the respondents’ sexual partners. HIV status disclosure was assessed among individuals who had previously received individual HCT (i.e. not with their sexual partners). Individuals who reported previous HIV status disclosure were asked if they and their current marital partners had ever disclosed their HIV status to each other. Marital order was grouped into three categories based on the number of marriages the respondent has had: first-order for those whose current marriage is the first ever, second-order for those who were in their second marriage, and third or higher for those who were in their third or higher order marriage.

### Data analysis

We conducted descriptive analysis to compute the characteristics of married individuals enrolled into the study and conducted inferential statistics to ascertain the correlates of previous couples’ HCT uptake among married individuals resident in each of the three HIV prevalence strata in Rakai, Uganda. At the bivariate analysis, we assessed the association between previous couples’ HCT uptake and each of the independent correlates (sociodemographic and behavioral characteristics, HIV status) and all variables with a *p*<0.2 (i.e. education, awareness that couples’ HCT services are available within the Rakai community cohort, and HIV status) and suspected confounders (i.e. age group, sex, marital duration, and marital order) were considered for the multivariable logistic regression model. At the multivariable analysis, we initially conducted stratified analyses to identify correlates associated with previous couples’ HCT within each stratum. However, the strata-based analyses yielded differing correlates with very wide confidence intervals (CIs). In the final model, we ran a combined model (using the same variables as in the stratified analysis); adjusting for clustering around HIV prevalence strata by using the *svyset* command in STATA. A *p*<0.05 was considered significant at the multivariable analysis level. Analysis was conducted using STATA statistical version 11.2.

### Ethical considerations

Previous to each interview, all respondents were read a detailed consent form that explained the objectives and purpose of the study, benefits and risks of participation in the study, confidentiality issues, and voluntary participation in the study among other issues. All respondents gave written informed consent previous to participating in the study. The study protocol was approved by the Higher Degrees, Research and Ethical Committee of Makerere University School of Public Health and the Uganda National Council for Science and Technology.

## Results

### Characteristics of the study population

Two thousand one hundred and thirty five married individuals were enrolled into the baseline study, representing approximately 69% of the targeted sample. Of these, 743 (34.8%) individuals were enrolled from the low HIV prevalence stratum, 775 (36.3%) individuals were enrolled from the middle HIV prevalence stratum, while 617 (28.9%) were enrolled from the high HIV prevalence stratum.

Table 1 shows the characteristics of the study population. Majority of the respondents were female (*n*=1,100, 51.5%), aged 25 years or older (*n*=1,819, 85.2%), had primary education (*n*=1,432, 67.1%), and had been married for five or more years (*n*=1,783, 83.5%). Majority of the respondents (*n*=1,410, 66%) were in the first marital order while 725 (34%) were in the second or higher marital order. The proportion of those in the second or higher marital order was higher in the high HIV prevalence stratum (*n*=284, 46%) followed by the middle (*n*=232, 29.9%) and low HIV prevalence stratum (*n*=209, 28.1%) in that order. Overall, 291 (13.6%) of married individuals reported engaging in extramarital relations. Extramarital relations were common in the high HIV prevalence stratum (*n*=93, 15.1%) but were slightly lower in the low (*n*=109, 14.7%) and middle (*n*=89, 11.5%) HIV prevalence strata.

**Table 1:** Characteristics of study respondents by HIV prevalence strata, Rakai, Uganda

		HIV prevalence strata		
		
Characteristic	Total *N*=2,135 (%)	Low HIV prevalence, *N*=743 (%)	Middle HIV prevalence, *N*=775 (%)	High HIV prevalence, *N*=617 (%)
Age group				
15–24 years	316 (14.8)	80 (10.8)	76 (9.8)	160 (25.9)
25–34 years	986 (46.2)	345 (46.4)	342 (44.1)	299 (48.5)
35+years	833 (39.0)	318 (42.8)	357 (46.1)	158 (25.6)
Sex				
Female	1,100 (51.5)	374 (50.3)	411 (53.0)	315 (51.0)
Male	1,035 (48.5)	369 (49.7)	364 (47.0)	302 (49.0)
Education level				
None	124 (5.8)	55 (7.4)	22 (2.8)	47 (7.6)
Primary	1,432 (67.1)	460 (61.9)	528 (68.1)	444 (72.0)
Post-primary	579 (27.1)	228 (30.7)	225 (29.0)	126 (20.4)
Marital duration				
1–2 years	132 (6.2)	21 (2.8)	14 (1.8)	97 (15.7)
3–4 years	220 (10.3)	44 (5.9)	44 (5.7)	132 (21.4)
5+years	1,783 (83.5)	678 (91.3)	717 (92.5)	388 (62.9)
Marital order				
First	1,410 (66.0)	534 (71.9)	543 (70.1)	333 (54.0)
Second	551 (25.8)	157 (21.1)	167 (21.5)	227 (36.8)
Third or more	174 (8.2)	52 (7.0)	65 (8.4)	57 (9.2)
Extramarital relations				
Yes	291 (13.6)	109 (14.7)	89 (11.5)	93 (15.1)
No	1,844 (86.4)	634 (85.3)	686 (88.5)	524 (84.9)
HIV status				
Negative	1,688 (79.1)	568 (76.4)	626 (80.8)	494 (80.1)
Positive	160 (7.5)	14 (1.9)	26 (3.3)	120 (19.4)
Not available	287 (13.4)	161 (21.7)	123 (15.9)	3 (0.5)
Knows that HIV-discordance is possible among couples				
Yes	2,113 (99.0)	735 (98.9)	769 (99.2)	609 (98.7)
No	22 (1.0)	8 (1.1)	6 (0.8)	8 (1.3)
Ever received HCT				
Yes	2,020 (94.6)	704 (94.7)	707 (91.2)	609 (98.7)
No	115 (5.4)	39 (5.3)	68 (8.8)	8 (1.3)
Time since last received HCT^a^				
Less than a year	1,359 (67.3)	384 (54.6)	377 (53.3)	598 (98.2)
1 year	235 (11.6)	127 (18.0)	99 (14.0)	9 (1.5)
2+years	426 (21.1)	193 (27.4)	231 (32.7)	2 (0.3)
Organization where HCT was received from				
Rakai health sciences program	1,891 (88.6)	641 (86.3)	647 (83.5)	603 (97.7)
Other organization	244 (11.4)	102 (13.7)	128 (16.5)	14 (2.3)
Aware of availability of couples’ HCT services				
Yes	2,060 (96.5)	705 (94.9)	752 (97.0)	603 (97.7)
No	75 (3.5)	38 (5.1)	23 (3.0)	14 (2.3)
HIV status disclosure (ever)^a^				
Yes	1,000 (85.2)	318 (80.5)	346 (84.0)	336 (91.6)
No	174 (14.8)	77 (19.5)	66 (16.0)	31 (8.4)

a Expressed among those who reported previous receipt of individual HCT.

When asked if they had ever heard that couples’ HCT services were available and provided within the Rakai community cohort, nearly all respondents (*n*=2,060, 96.5%) reported that they were aware that these services were available, with no significant differences observed across HIV prevalence strata. Awareness that a couple can have HIV-discordant status, that is, that one partner can be HIV positive while the other partner is HIV negative, was universal (*n*=2,113, 99%). Nearly all respondents (*n*=2,020, 95%) had ever received their HIV test results (regardless of whether or not they received them together with their partners); majority (*n*=1,891, 87%) reported that they had ever received their HIV test results from the Rakai Health Sciences Program. Slightly over two-thirds (*n*=1,359, 67.3%) of those that had ever received HCT reported that they last received them within 12 months preceding the interview.

Overall, 1,174 (58.1%) of the ever-tested individuals reported that they had ever received individual HCT (i.e. alone) rather than together with their partners. Of these, 1,000 (85.2%) reported that they had ever disclosed their HIV status to any of their sexual partners. The proportion of those who had ever disclosed their HIV status to any of their sexual partners increased significantly with increasing HIV prevalence levels from 318 (80.5%) in the low HIV prevalence stratum to 346 (84%) in the middle HIV prevalence stratum and 336 (91.6%) in the high HIV prevalence stratum (*p*<0.0001). Of those that had ever disclosed their HIV status to any of their sexual partners, 494 (49.4%) reported that they disclosed their HIV status to their current marital partners in the past 12 months preceding the interview.

### Previous couples’ HIV counseling and testing uptake

Table 2 shows previous receipt of couples’ HCT among 2,020 ever-tested individuals stratified by background characteristics and HIV prevalence strata. Of these, 846 (41.9%) reported that they had ever received couples’ HCT. There was no significant difference in the proportion of those who had ever received couples’ HCT in the low (*n*=309, 43.9%), middle (*n*=295, 41.7%), and high (*n*=242, 39.7%) HIV prevalence settings (*p*=0.61). Of those that had ever received couples’ HCT, 802 (94.8%) reported that they had ever received couples’ HCT with their current marital partners. The proportion of those who had ever received couples’ HCT with their current marital partners was higher in the middle HIV prevalence stratum (*n*=284, 96.3%) followed by those in the high HIV prevalence strata (*n*=228, 94.2%) and low HIV prevalence strata (*n*=290, 93.8%) in that order.

**Table 2:** Previous receipt of couples’ HIV counseling and testing among ever-tested married individuals by background characteristics and HIV prevalence strata, Rakai, Uganda

			HIV prevalence strata					
			
	Overall		Low HIV prevalence		Middle HIV prevalence		High HIV prevalence	
	
Characteristic	Ever-tested individuals (*N*)	Number and proportion^a^ with previous couples’ HCT	Ever-tested individuals (*N*)	Number and proportion^a^ with previous couples’ HCT	Ever-tested individuals (*N*)	Number and proportion^a^ with previous couples’ HCT	Ever-tested individuals (*N*)	Number and proportion^a^ with previous couples’ HCT
All tested individuals	2,020	846 (41.9)	704	309 (43.9)	707	295 (41.7)	609	242 (39.7)
Age group								
15–24 years	309	127 (41.1)	77	33 (42.9)	73	31 (42.5)	159	63 (39.6)
25–34 years	939	400 (42.6)	325	144 (44.3)	318	136 (42.8)	296	120 (40.5)
35 + years	772	319 (41.3)	302	132 (43.7)	316	128 (40.5)	154	59 (38.3)
Education level								
None	118	51 (43.2)	52	31 (59.6)	20	7 (35.0)	46	13 (28.3)
Primary	1,341	537 (40.0)	433	178 (41.1)	469	191 (40.7)	439	168 (38.3)
Post-primary	561	258 (46.0)	219	100 (45.7)	218	97 (44.5)	124	61 (49.2)
Sex								
Female	1,071	447 (41.7)	366	162 (44.3)	393	160 (40.7)	312	125 (40.1)
Male	949	399 (42.0)	338	147 (43.5)	314	135 (43.0)	297	117 (39.4)
HIV status (*N*=1,763)								
Negative	1,613	681 (42.2)	541	233 (43.1)	581	245 (42.2)	491	203 (41.3)
Positive	150	54 (36.0)	13	8 (61.5)	22	8 (36.4)	115	38 (33.0)
Aware about availability of couples’ HCT services								
Yes	1,950	840 (43.1)	667	306 (45.9)	688	293 (42.6)	595	241 (40.5)
No	70	06 (8.6)	37	3 (8.1)	19	2 (10.5)	14	1 (7.1)
Knows that couples can have HIV-discordant status								
Yes	2,000	840 (42.0)	696	306 (44.0)	703	293 (41.7)	601	241 (40.1)
No	20	6 (30.0)	8	3 (37.5)	4	2 (50.0)	8	1 (12.5)
Marital duration								
1–2 years	129	51 (39.5)	18	9 (50.0)	14	5 (35.7)	97	37 (38.1)
3–4 years	213	89 (41.8)	42	24 (57.1)	40	17 (42.5)	131	48 (36.6)
5 + years	1,678	706 (42.1)	644	276 (42.9)	653	273 (41.8)	381	157 (41.2)
Marital order								
First order	1,343	558 (41.6)	512	213 (41.6)	502	210 (41.8)	329	135 (41.0)
Second order	518	213 (41.8)	144	68 (47.2)	150	57 (38.0)	224	88 (39.3)
Third order or higher	159	75 (47.2)	48	28 (58.3)	55	28 (50.9)	56	19 (33.9)
Extramarital relations								
Yes	264	105 (39.8)	100	38 (38.0)	73	30 (41.1)	91	37 (40.7)
No	1,756	741 (42.2)	604	271 (44.9)	634	265 (41.8)	518	205 (39.6)
Study arm								
Comparison	884	407 (46.0)	228	99 (43.4)	433	200 (46.2)	223	108 (48.4)
Intervention	1,136	439 (38.6)	476	210 (44.1)	274	95 (34.7)	386	134 (34.7)

a Proportion of individuals who received previous couples’ HCT among those who have ever received HCT.

Table 2 also shows that respondents who were aware that couples’ HCT services were provided within the Rakai community cohort (*n*=1,950, 43.1%) were significantly more likely to report previous receipt of couples’ HCT than those who were not (*n*=70, 8.6%, *p*<0.0001). However, there was no significant difference in previous receipt of couples’ HCT between those who knew that couples can have HIV-discordant results (*n*=2,000, 42%) and those who did not (*n*=20, 30%, *p*=0.23).

Previous receipt of couples’ HCT did not differ by age group, education level, sex, HIV status, marital duration, or marital order. However, although there was no significant association between marital duration and previous receipt of couples’ HCT (*p*=0.94) at the bivariate analysis, previous receipt of couples’ HCT seemed to increase with increasing marital duration from 129 (39.5%) among those who had stayed together for 1–2 years to 213 (41.8%) among those who had stayed together for 3–4 years and 1,678 (42.1%) among those who had been together for five or more years.

### Correlates of previous couples’ HIV counseling and testing uptake

In the initial strata-stratified analyses, we did not find any correlates that were significantly associated with previous receipt of couples’ HCT across the three HIV prevalence strata. Instead, different correlates were associated with previous receipt of couples’ HCT in different HIV prevalence strata, usually with very wide CIs. Due to these differences, we decided to run a combined model that accounted for clustering around HIV prevalence strata as shown in Table 3. The results of the combined model indicated that individuals who had stayed together for five or more years [adjusted odds ratio (aOR): 1.06; 95% CI: 1.04–1.08] and those that were aware of the availability of couples’ HCT services within the Rakai Community Cohort (aOR=7.58, 95% CI: 5.63–10.20) were significantly more likely to report previous receipt of couples’ HCT than their counterparts.

**Table 3:** Unadjusted and adjusted^a^ odds ratios and 95% CIs associated with previous receipt of couples’ HCT among 1,848 individuals with known HIV status

Variable	Unadjusted odds ratios (95% CI)	Adjusted odds ratios (95% CI)
Sex		
Female	1.00	1.00
Male	0.90 (0.75–1.09)	0.91 (0.70–1.18)
Age group		
15–24 years	1.00	1.00
25–34 years	1.02 (0.78–1.35)	1.02 (0.76–1.37)
35+years	0.97 (0.73–1.29)	0.94 (0.55–1.61)
Education level		
None	1.00	1.00
Primary	0.87 (0.58–1.30)	0.86 (0.15–5.01)
Post-primary	1.16 (0.76–1.77)	1.14 (0.16–7.86)
Awareness about the availability of couples’ HCT services		
No	1.00	1.00
Yes	7.88 (2.82–21.98)	7.58 (5.63–10.20)*
HIV status		
HIV negative	1.00	1.00
HIV positive	0.75 (0.53–1.06)	0.75 (0.54–1.06)
Marital order		
First	1.00	1.00
Second	0.96 (0.77–1.19)	1.09 (0.84–1.40)
Third or higher	1.12 (0.79–1.58)	1.32 (0.47–3.68)
Marital duration		
1–2 years	1.00	1.00
3–4 years	1.00 (0.63–1.58)	1.00 (0.53–1.90)
5+years	1.07 (0.74–1.56)	1.06 (1.04–1.08)**

CI=confidence interval.

a Adjusted for education level, awareness about the existence of couples’ HCT services within the Rakai cohort, HIV status, sex and age group, marital order and marital duration. The OR and 95% CI have been adjusted for clustering around HIV prevalence strata.

* *p*=0.001

** *p*=0.006

## Discussion

Our study of previous couples’ HCT uptake in Rakai, Uganda, shows that almost one in two married individuals have ever received their HIV test results together as a couple. Previous receipt of couples’ HCT was significantly higher among those who had been living together for five or more years and those who were aware of the existence of couples’ HCT services within the Rakai Community Cohort. Individuals living together for a longer duration have been found to report higher rates of HIV status disclosure (19); thus, the finding that longer duration was associated with previous receipt of couples’ HCT may imply that living together for a longer duration predicts acceptance of couples’ HCT. Of concern, though, is the fact that individuals in newer relationships (i.e. those with a shorter duration of marriage) were less likely to report previous couples’ HCT. Considering that the risk of HIV infection may be higher in newer relationships (20), these findings call for a need to emphasize the importance of couples’ HIV testing and joint awareness of HIV status prior to or immediately after marital formation.

The finding that those who were aware of the existence of couples’ HCT services were more likely to report previous couples’ HCT than those who were not suggests that informing couples about the existence of HCT services in their community can influence uptake of couples’ HCT services (21, 22). A recent study among commuters in South Africa found that awareness of HCT services improved the likelihood of HIV testing (23), further emphasizing the importance of creating awareness about HCT services availability in the community. However, it is important to note that while awareness of the availability of couples’ HCT services was nearly universal (97%), only 43% of couples that were aware of these services reported previous receipt of couples’ HCT. This means that mere awareness of the availability of services may not necessarily increase couples’ HCT uptake (22), suggesting a need for more aggressive demand-creation interventions that not only increase awareness about services availability but also address the apparent fears and reluctance among couples to receive couples’ HCT (11, 24).

We found no significant difference in previous receipt of couples’ HCT between low-, middle-, and high HIV prevalence settings. The apparent reasons for this finding are not clear, warranting a need for further inquiry. However, uptake of previous couples’ HCT was slightly higher in low HIV prevalence settings than in high HIV prevalence settings, suggesting a need for strata-specific interventions. For instance, since individuals in high HIV prevalence settings were significantly more likely to report HIV status disclosure than those in low HIV prevalence settings, it is likely that interventions that promote counselor-assisted HIV status disclosure (25) in these settings can increase the proportion of married individuals that are aware of each other's HIV status. On the contrary, since individuals in low- and medium HIV prevalence settings reported higher uptake of couples’ HCT than those in high HIV prevalence settings, it is likely that promotion of couples’ HCT uptake may be a more acceptable approach for increasing the proportion of married individuals that are aware of each other's accurate HIV status (10).

We have reported that only 42% of married individuals had ever received HCT as a couple. Couples’ HCT services have been available in the Rakai Community Cohort since 1994 (15, 26) and these services are provided free of cost. Despite this availability, nearly 6 of every 10 couples in the Rakai Community Cohort have never tested as a couple. Our previous findings show that individuals are more likely to test individually than together with their partners (15), and that fear of the consequences of receiving couples’ HCT (11) remain key barriers to couples’ HCT uptake in this cohort. This study was conducted to generate data necessary to inform the design of a community-based, demand-creation intervention aimed at improving couples’ HCT uptake among married couples in Rakai, Uganda. We anticipate that this intervention will increase the proportion of married individuals that receive couples’ HCT services, and who can then be linked to appropriate HIV prevention, care and treatment services.

The findings of this study should be interpreted with caution. There is a possibility that the reported couples’ HCT uptake rates might not reflect the actual uptake rates in the community especially if, in reporting about the previous uptake, individuals who had ever received couples’ HCT with previous and current partners forget to report about receipt with both partners. However, this is less likely to affect the reported rates considering that a general question (‘Have you ever received couples’ HCT with any of your sexual partners?’) was administered before the respondent was asked about previous couples’ HCT with the current partner. In any case, 95% of those who reported previous couples’ HCT also reported that they have ever received couples’ HCT with their current marital partners. Thus, it is likely that the uptake rates reported reflect the true picture of couples’ HCT uptake in this cohort. It is also important to note that the HIV status disclosure rates reported in this paper are largely based on individual self-reports and might not reflect the true HIV status disclosure rates in the community. Nevertheless, our findings are consistent with previous findings in Uganda and elsewhere (9, 27), suggesting that they are generalizable to married individuals in other settings.

## Conclusion

Our study shows that previous couples’ HCT uptake did not differ by HIV prevalence settings. However, we found that longer duration in marriage and awareness of the existence of couples’ HCT services in the community were significantly associated with previous receipt of couples’ HCT services in this cohort. These findings suggest a need for innovative demand-creation interventions that not only increase awareness about service availability but also address other barriers to couples’ HCT in order to improve uptake of couples’ HCT services.
